# Use of population health data to promote equitable recruitment for a primary care practice implementation trial addressing unhealthy alcohol use

**DOI:** 10.1017/cts.2023.530

**Published:** 2023-04-14

**Authors:** Alex H. Krist, Alison N. Huffstetler, Gabriela Villalobos, Michelle S. Rockwell, Alicia Richards, Adam Funk, Roy T. Sabo, Beth Bortz, Ben Webel, Jong Hyung Lee, Kyle Russel, Anton Kuzel, Jaqueline B. Britz, F. Gerard Moeller

**Affiliations:** 1 Department of Family Medicine and Population Health, Virginia Commonwealth University, Richmond, VA, USA; 2 Inova Health System, Fairfax, VA, USA; 3 Wright Center for Clinical and Translational Research, Virginia Commonwealth University, Richmond, VA, USA; 4 Department of Family & Community Medicine, Virginia Tech Carilion School of Medicine, Roanoke, VA, USA; 5 Department of Biostatistics, Virginia Commonwealth University, Richmond, VA, USA; 6 Virginia Center for Health Innovation, Richmond, VA, USA; 7 Virginia Health Information, Richmond, VA, USA

**Keywords:** Prevention, equitable recruitment, real-world data, primary care, unhealthy alcohol use

## Abstract

**Background:**

Recruiting underrepresented people and communities in research is essential for generalizable findings. Ensuring representative participants can be particularly challenging for practice-level dissemination and implementation trials. Novel use of real-world data about practices and the communities they serve could promote more equitable and inclusive recruitment.

**Methods:**

We used a comprehensive primary care clinician and practice database, the Virginia All-Payers Claims Database, and the HealthLandscape Virginia mapping tool with community-level socio-ecological information to prospectively inform practice recruitment for a study to help primary care better screen and counsel for unhealthy alcohol use. Throughout recruitment, we measured how similar study practices were to primary care on average, mapped where practices’ patients lived, and iteratively adapted our recruitment strategies.

**Results:**

In response to practice and community data, we adapted our recruitment strategy three times; first leveraging relationships with residency graduates, then a health system and professional organization approach, followed by a community-targeted approach, and a concluding approach using all three approaches. We enrolled 76 practices whose patients live in 97.3% (1844 of 1907) of Virginia’s census tracts. Our overall patient sample had similar demographics to the state for race (21.7% vs 20.0% Black), ethnicity (9.5% vs 10.2% Hispanic), insurance status (6.4% vs 8.0% uninsured), and education (26.0% vs 32.5% high school graduate or less). Each practice recruitment approach uniquely included different communities and patients.

**Discussion:**

Data about primary care practices and the communities they serve can prospectively inform research recruitment of practices to yield more representative and inclusive patient cohorts for participation.

## Background

The lack of equitable representation in research compounds health inequities and has serious consequences [1]. Both mistrust and structural problems in how we conduct research to contribute to lack of representation in research, underrepresented people, and communities are generally willing to participate in research if respectfully asked.

A key function of Clinical Translational Science (CTS) Centers is to build infrastructure and methods that promote equitable inclusion of socially and economically marginalized and medically underserved people and communities in translational research [2]. Nationally, CTS centers are using novel community engagement methods to design more acceptable and feasible research, build relationships and establish trust, and work to ensure that research aligns with the needs of vulnerable and underrepresented people [3]. CTS Centers’ success is partially judged by their ability to promote more inclusive and representative participation in research [4,5]. The novel integration of population health data can further add to CTS Centers’ ability to design and recruit for more equitable research.

In 2019, the Agency for Healthcare Research and Quality funded six grantees to provide practice facilitation to 125 primary care practices to better implement the U.S. Preventive Services Task Force (USPSTF) recommendation to screen for unhealthy alcohol use and brief counseling and treatment for patients with hazardous drinking [6]. This was done through the EvidenceNow initiative, a series of funding announcements to promote the uptake of guidelines and evidence-based practice into routine care [7].

Unhealthy alcohol use is the third leading cause of preventable death in the USA [8]. There is strong evidence to demonstrate the benefits of screening and counseling for unhealthy alcohol use in primary care [9], hence the USPSTF recommends that primary care clinicians provide this service routinely for all adults [10]. While highly effective and feasible to deliver in primary care—taking one to two sessions and 2–30 thirty minutes in implementation trials reviewed by the USPSTF—these services are poorly delivered. Most clinicians report that they do not routinely screen or provide counseling and treatment interventions, and most patients do not recall being asked about alcohol use or receive feedback on their drinking habits [11–14].

Despite the need and value of promoting this preventive service, enrolling 125 busy primary care practices for training to better deliver under-delivered services is an audacious task that is reliant on established relationships with practices. All grantees in this initiative were primarily CTS centers and practice-based research networks. Adding to the complexity of recruiting such a large practice sample, shortly after this initiative started the COVID pandemic hit. Primary care practices—already in survival mode—eschewed any task that was not essential to addressing COVID or their core responsibilities, like participating in research [15]; concurrently, the COVID pandemic made unhealthy alcohol use worse, further intensifying the need for these services. Prior to the pandemic 20–25% of US adults drank at unhealthy levels and 14% had alcohol use disorder, and these rates substantially increased during the pandemic [16,17].

This manuscript describes the novel use of a comprehensive primary care clinician and practice database, the Virginia All-Payers Claims Database (APCD), and an analytic database and mapping tool with community-level socio-ecological information, called HealthLandscape Virginia (HLVA), to ensure successful and inclusive recruitment of primary care practices for an implementation study to address unhealthy alcohol use [18–20].

## Methods

This is a prospective observational analysis of the recruitment strategy for a practice-level randomized controlled trial to provide early versus delayed practice facilitation for improving screening for unhealthy alcohol use and brief counseling interventions for hazardous drinking. We prospectively analyzed the inclusiveness of the practices and communities recruited for participation and iteratively adapted our recruitment strategies to ensure all communities in Virginia would be represented and benefit.

Practices were approached to participate in this study and provided the details of the intervention. Specifically, practices were randomized to immediate versus 6-month delayed practice facilitation. Each practice was asked to identify one to three practice champions to participate in up to four learning collaboratives to develop a standard approach to implementing the screening and counseling recommendation. Then the champions led up to three practice-wide sessions to implement the screening and counseling workflow. Champions and practices had access to a board spectrum of patient and community-level resources [21]. We conducted a chart review and mailed a survey to 60 patients randomly selected at baseline, 3 months, and 6 months postintervention to assess the impact of the practice facilitation intervention. This manuscript focuses on the use of baseline results for recruitment.

The methods for the practice-level pragmatic trial have been reported previously [22]. This study was approved by the Virginia Commonwealth Institutional Review Board (HM20016728).

**Recruitment Approach**. The EvidenceNow Request for Applications for this funding initiative (RFA-HS-18-002) defined recruitment of 125 practices and providing practice facilitation for screening and counseling for unhealthy alcohol use as a key requirement for applicants and the primary measure of success [23]. To address this requirement, in our proposal we described a practice recruitment strategy that would leverage existing relationships and connections with our primary care practice-based research network, the Virginia Ambulatory Care Outcomes Network (ACORN), and our five Virginia Commonwealth University and VirginiaTech Carilion family medicine residency training centers. Both ACORN and the residencies are distributed throughout the state and approximately 40% of graduating residents continue to practice in Virginia after matriculation. We defined five regional hubs centered around each residency to recruit 30 local practices for participation; we planned to oversample in the event practices dropped out of the study.

With the onset of the COVID pandemic, we quickly realized that the stresses experienced by our training centers and ACORN network practices would make it difficult to meet the required 125 practices for participation. We also realized that the practices initially recruited were not fully representative of primary care or the communities primary care practices serve in Virginia, so we created a process to use real-world data to improve and monitor recruitment. This process used a novel internal database of all primary care practices and clinicians in Virginia to identify the practices participating in our study and who could be recruited for participation, the APCD to identify the communities impacted by practices participating of not participating, and HLVA to visually and geographically understand the characteristics of the communities included and not included in our study.

This resulted in four unique recruitment approaches:

- Approach 1 (*Training Approach*) – as described in our proposal, we created five hubs at the residency practices throughout the state. The residencies participated in the study and a faculty champion contacted graduates and local practices about study participation.
- Approach 2 (*Health System and Professional Organization Approach*) – we recruited leadership from health systems in Virginia who agreed to disseminate recruitment information to their practices. The Virginia primary care specialty societies agreed to send recruitment material to their membership.
- Approach 3 (*Targeted Approach*) – using the APCD and primary care database, we identified primary care practices in communities with low inclusion from prior recruitment efforts and emailed, mailed, or called the practices about study participation. These practices were not part of health systems that agreed to participate in Approach 2 and had not participated in prior ACORN studies.
- Approach 4 (*Concluding Approach*) – we repeated the actions in Approaches 1–3.

**Outcomes**. The outcomes for this analysis include the number of practices recruited using each approach and the characteristics of the practices, communities, and patients included in the study for each recruitment approach.

**Data Sources**. We used four data sources to prospectively inform our recruitment process and to assess the outcomes of the four recruitment approaches. The first data source is the ACORN primary care clinician and practice registry for Virginia. This data source is supported and maintained by ACORN, the Wright Center for Clinical and Translational Research, and the Department of Medical Assistance Services to inform ACORN activities, support translational research efforts, and track the role of primary care with Medicaid Expansion [24,25]. The methods used to create the database have been described previously [26]. To identify the primary care workforce, we annually query the National Plan and Provider Enumeration System to identify specialty, verify the presence of an active claim in the APCD, and verify at least 10 wellness visits for nonfamily medicine clinicians in the APCD. We identify new clinicians, any change in claims activity (i.e., absence of claims or new wellness claims for clinicians), or any change in location compared to what is currently in the ACORN database. Clinician and practice data are verified manually with online information.

The second data source is Virginia’s APCD. We use this to identify the primary care clinician workforce (described above) and to identify the census tract that patients live in for the participating practices. Virginia has a robust and comprehensive APCD. It was established in 2013 and contains data on roughly 65% (5.5 million out of 8.5 total) of Virginians, including 100% of Medicare and Medicaid claims and 50% of commercial claims [27]. The third data source is the HLVA Community Vital signs library [20]. This includes census tract-level variables on a range of socioecological factors such as poverty, education, race/ethnicity, internet access, transportation, housing foreclosures and vacancies, residential segregation, small area measures of chronic conditions, access to care, inequality, and composite measures of deprivation and vulnerability (social deprivation index, social vulnerability index, and index of deep disadvantage). Data come from multiple sources such as the American Community Survey, 500 Cities Project, Behavioral Risk Factor Surveillance System, National Health and Nutrition Examination Survey, and more [28–30]. The demographic data used as the comparison for our analysis was primarily from the American Community Survey. It describes the demographics for Virginians of all ages, except marital status and education are for residents ages 15 years and older (note our study sample is patients ages18–79 years).

The final data source is the chart review and patient survey responses for baseline patients included in the practice facilitation trial. At enrollment, each practice generated a list of patients aged 18 to 79 years seen in the prior 3 months that would be eligible to receive screening and counseling for unhealthy alcohol use. We randomly selected 60 patients for inclusion and our research coordinator conducted a chart review to assess demographic characteristics (age, sex, race, ethnicity, insurance type, and preferred language). The same patients were also mailed a survey using a modified Dillman technique to assess demographic information not routinely documented in the medical record, such as marital status and education, as well as outcomes from our clinical trial.

**Analysis.** We compared the practice characteristics of enrolled practices to all the practices in the Virginia primary care database, and the patient characteristics from the chart review and patient survey to the overall demographics of Virginia using the 2021 American Community Survey and 2022 Medicaid and CHIP data [31,32]. Additionally, practice rurality was determined using the patient zip codes and the locale codes created by the Education Demographic and Geographic Estimates (EDGE) Program [33]. We defined each overall practice and patient characteristic as similar to the state average if the value was between 80% and 120% of the state average. Likewise, we defined the practice and patient characteristics for each of the four approach periods as similar to the overall study sample if its value was between 80% and 120% of the overall study sample average. To map the communities potentially impacted by our study, we used the APCD to identify each census tract where one or more patients lived that was seen in the recruited practice in 2019. Practice and patient characteristics were summarized with frequencies and percentages. Chi-squared tests and Fisher’s exact tests were used to determine if there were differences in characteristics across approaches.

The GIS software ArcGIS Pro 3.0.3, developed by the Environmental Systems Research Institute (ESRI), was used for creating maps. We first imported and created a geodatabase in ArcGIS that contained a specific approach (i.e., approaches 1–4 and overall patient density (per 2,500)) and the corresponding patients for each census tract within Virginia.

We then conducted a spatial join between this geodatabase and the shapefile of Virginia based on the unique census tract field (U.S. Census Bureau, 2019). Since the number of patients was heavily right skewed for all approaches, we manually defined classifications, though for easier comparison, we incorporated the same cutoff values for the maps of approaches 1 through 4 [34]. All statistical analyses were performed at a significance level of 0.05 using R version 4.1.0.

## Results

**Figure 1.**
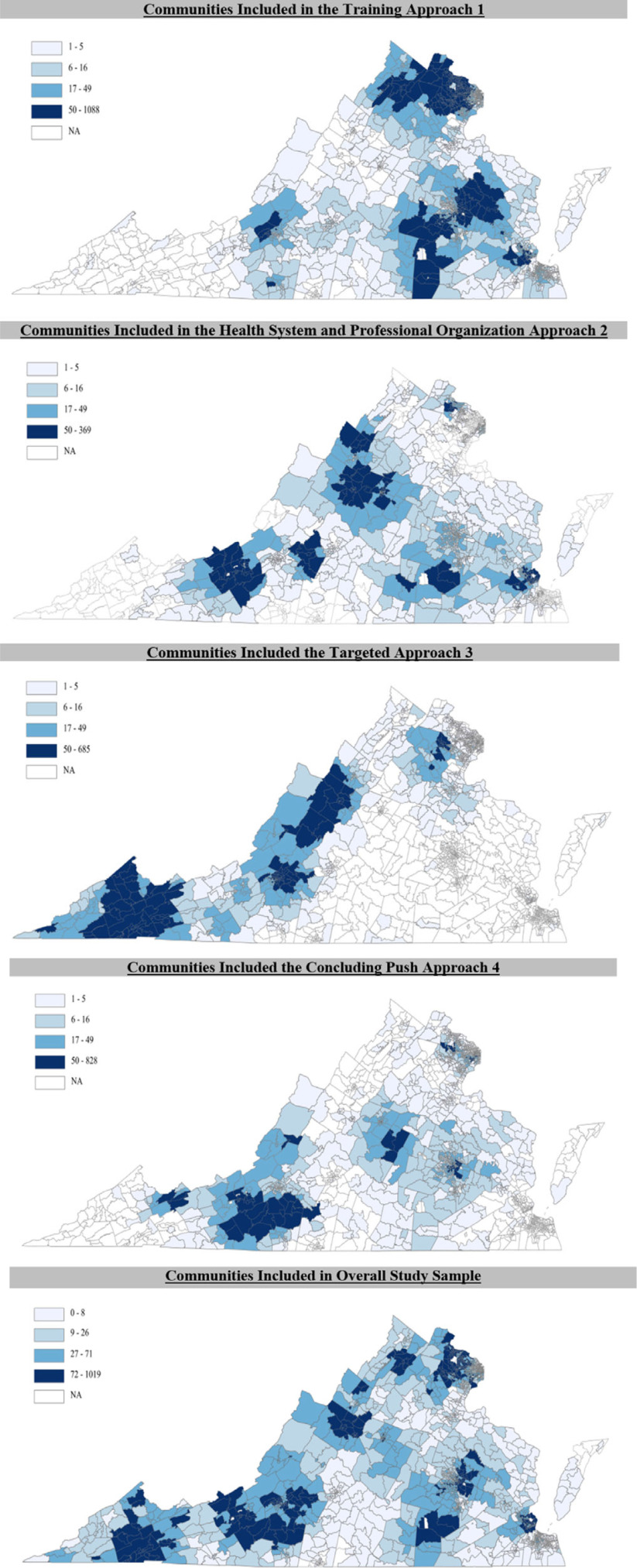
Distribution of where the study practices’ patients live. these maps show the number of people that live in each census tract of virginia that had an office visit with one of the recruited study practices in each of the four recruitment approaches and for the overall study sample.

Using our four recruitment approaches, we enrolled 76 practices for study participation (Table 1). The residency training approach (#1) enrolled 24 practices with patients living in 1681 census tracts; the health system approach (#2) enrolled 17 practices with patients living in 1126 census tracts; the targeted approach (#3) enrolled 22 practices with patients living in 892 census tracts; and the final push approach (#4) enrolled 13 practices living in 1201 census tracts. As a result, the final practice sample cared for patients living in 97.3% (1844 of 1907) of Virginia’s census tracts (Fig. 1).

**Table 1.** Characteristics of practices enrolled in the study

Practice characteristic	Practices in training approach 1 (*n* = 24)	Practices in system approach 2 (*n* = 17)	Practices in targeted approach 3 (*n* = 22)	Practices in concluding approach 4 (*n* = 13)	All practices in study (*n* = 76)	All adult primary care practices in virginia (*n* = 2,026)^a^	*p* value
Practice type family/internal medicine	23 (95.8%)	12 (70.6%)	19 (96.4%)	9 (69.2%)	63 (82.9%)	358 / 445 (80.4%)	0.029^b^
Obstetrics and gynecology	0 (0%)	1 (5.9%)	0 (0%)	3 (23.1%)	4 (5.3%)	20 / 445 (4.5%)	
Mixed primary/specialty	1 (4.2%)	4 (23.5%)	3 (13.6%)	1 (7.7%)	9 (11.8%)	67 / 445 (15.1%)	
Practice location Urban	4 (16.7%)	3 (17.7%)	1 (4.6%)	3 (23.1%)	11 (14.5%)	454 / 2022 (22.5%)	0.157^b^
Suburban	11 (45.8%)	2 (11.8%)	7 (31,8%)	4 (30.8%)	24 (31.6%)	645 / 2026 (31.9%)	
Rural	9 (37.5%)	12 (70.6%)	14 (63.6%)	6 (46.2%)	41 (54.0%)	923 / 2026 (45.6%)	
Practice ownership University	0 (0%)	1 (5.9%)	0 (0%)	0 (0%)	1 (1.3%)	5 / 429 (1.2%)	0.004^b^
Health system	13 (54.2%)	9 (52.9%)	21 (95.5%)	11 (84.6%)	54 (71.1%)	181 / 429 (42.2%)	
Clinician	9 (37.5%)	3 (17.6%)	1 (4.5%)	2 (15.4%)	15 (19.7%)	161 / 429 (37.5%)	
Private sponsor/corporation	0 (0%)	0 (0%)	0 (0%)	0 (0%)	0 (0%)	77 / 429 (17.9%)	
Community health center	2 (8.3%)	4 (23.5%)	0 (0%)	0 (0%)	6 (7.9%)	5 / 429 (1.2%)	
Patient Centered Medical Home designation	12 (50%)	9 (52.9%)	14 (63.6%)	5 (38.5%)	40 (52.6%)	132 / 423 (31.2%)	0.535^b^
Part of an Accountable Care Organization	16 (66.7%)	7 (41.2%)	18 (81.8%)	7 (53.8%)	48 (63.2%)	183 / 410 (44.6%)	0.058
Direct primary care practice	1 (4.2%)	0 (0%)	0 (0%)	1 (7.7%)	2 (2.6%)	58 / 429 (12.8%)	0.540^b^
Number unique census tracts that practices’ patients live in	1681	1126	892	1201	1844	--	--
Number of new census tracts that practices’ patients live in compared to prior approaches	1681	89	60	3	--	--	--

*p* value compares the practice demographics from wave to wave using a two-tailed *t* test.

For *Approach #1–4* cells, green is 20% higher than *All Practice* characteristic and orange is 20% lower.

For *All Practices* cells, green is 20% higher than *All Practices in Virginia* and orange is 20% lower.

a Percentages based on responses to the Virginia Primary Care Survey responses [18]. The survey was mailed to Virginia’s 2,296 primary care practices; 445 of 2,026 practices for adults (22%) completed the survey.

b Fisher Exact test was used as Chi-squared was not sufficient.

The practices enrolled for study participation were generally representative of primary care in Virginia with some important exceptions (Table 1). Compared with all of Virginia’s primary care practices, the practices participating in our study were less likely to be urban (14.5% vs 22.5%), more likely to be health system owned (71.1% vs 42.2%) or a community health center (7.9% vs 1.2%), and more likely to be academically oriented including patient-centered medical home designation (52.6% vs 63.1%) or part of an accountable care organization (63.2% vs 44.6%). Across approaches there were significant differences in practices enrolled between practice type (*p* = 0.029) and practice ownership (*p* = 0.004). The training approach recruited more suburban and clinician-owned practices than the other approaches; the health system and professional organization approach more mixed primary-specialty care, urban, rural, and community health center practices; the targeted approach more unique practices from nonrepresented health system and academically oriented practices; and the concluding approach added some obstetrics-gynecology and direct primary care practices.

The characteristics of the overall sample of study patients were very similar to the demographics of Virginia with respect to sex, ethnicity, race, commercial or Medicaid insurance status, and education (Table 2). Our sample only differed by including fewer patients who were Asian (5.7% vs 7.2%), whose preferred language was Spanish (4.2% vs 7.1%) or a language other than English (1.4% vs 8.3%), had Medicaid (13.7% vs 22.5% or Tricare (0.9% vs 1.8%) for insurance, had a high school education (19% vs 23.9%), or were never married (18.9% vs 32%) or were separated 3.7% vs 5.5%). Across recruitment waves, there were statistically significant differences in ethnicity (*p* < 0.001), race (*p* < 0.001), language (*p* < 0.001), insurance (*p* < 0.001), education (*p* < 0.001), and marital status (*p* < 0.001). The training approach recruited more Black, Asian, and educated patients; the health system and professional organization approach more patients with Medicaid insurance and never married or divorced people; the targeted approach more Hispanic, Spanish or other language speaking, self-pay and uninsured, and less than high school educated patients; and the concluding approach more patients with Medicaid insurance and other language speaking (Table 2).

**Table 2.** Demographic characteristics of patients included in the study^**a**^

Characteristic	Patients in training approach 1 (*n* = 1363)	Patients in system approach 2 (*n* = 1017)	Patients intargeted approach 3 (*n* = 900)	Patients in concluding approach 4 (*n* = 600)	All patients in study (*n* = 3,880)	Virginia residents^b^ (*n* = 8,657,365)	*p* value^c^
Sex^d^ female	821/1363 (60.2%)	618/1017 (60.8%)	532/900 (59.1%)	377/600 (62.8%)	2348/3880 (60.5%)	50.5%	0.539
Ethnicity^d^ hispanic	47/996 (4.7%)	35/982 (3.6%)	219/879 (24.9%)	22/573 (3.8%)	323/3414 (9.5%)	10.2%	< 0.001
Race^d^ White	734/1214 (60.5%)	731/994 (73.5%)	582/815 (71.4%)	406/583 (69.6%)	2453/3606 (68.0%)	68.8%	<0.001
Black	371/1214 (30.6%)	162/994 (16.3%)	99/815 (12.2%)	120/583 (20.6%)	752/3606 (20.9%)	20.0%	
Asian	89/1214 (7.3%)	58/994 (5.8%)	28/815 (3.4%)	32/583 (5.5%)	207/3606 (5.7%)	7.2%	
Other	20/1214 (1.7%)	43/994 (4.3%)	106/815 (13.0%)	25/583 (4.3%)	194/3606 (5.4%)	3.9%	
Language^d^ english	1136/1166 (97.4%)	986/1006 (98.0%)	742/893 (83.1%)	593/599 (99.0%)	3457/3664 (94.4%)	84.6%	<0.001
Spanish	18/1166 (1.5%)	7/1006 (0.01%)	127/893 (14.2%)	3/599 (0.01%)	155/3664 (4.2%)	7.1%	
Other	12/1166 (0.01%)	13/1006 (1.3%)	25/893 (2.8%)	32/599 (5.3%)	52/3664 (1.4%)	8.3%	
Insurance^d^ commercial	924/1363 (67.9%)	550/1017 (54.1%)	395/900 (43.9%)	335/600 (55.8%)	2205/3880 (56.8%)	49.2%	<0.001
Medicaid	100/1363 (7.3%)	183/1017 (18.0%)	140/900 (15.6%)	109/600 (18.2%)	532/3880 (13.7%)	22.5% ^f^	
Medicare	285/1363 (20.9%)	243/1017 (23.9%)	193/900 (21.4%)	138/600 (23.0%)	859/3880 (22.1%)	18.5%	
Self-pay / uninsured	32/1363 (2.3%)	36/1017 (3.5%)	165/900 (18.3%)	16/600 (2.7%)	249/3880 (6.4%)	8.0%	
Tricare	21/1363 (1.5%)	5/1017 (0.5%)	7/900 (0.8%)	2/600 (0.3%)	35/3880 (0.9%)	1.8%	
Education^e^ less than HS	19/359 (5.3%)	13/252 (5.2%)	20/150 (13.3%)	8/95 (8.4%)	60/856 (7.0%)	8.6%	<0.001
HS grad / GED	66/359 (18.3%)	51/252 (20.2%)	28/150 (18.7%)	18/95 (18.9%)	163/856 (19.0%)	23.9%	
Some college	63/359 (17.5%)	82/252 (32.5%)	42/150 (28.0%)	27/95 (28.4%)	214/856 (25.0%)	25.7%	
College degree	120/359 (33.4%)	61/252 (24.2%)	39/150 (26.0%)	25/95 (26.3%)	245/856 (28.6%)	23.5%	
Graduate degree	91/359 (25.3%)	45/252 (17.9%)	21/150 (14.0%)	17/95 (17.9%)	174/856 (20.3%)	18.3%	
Marital status^e^ never married	66/362 (18.2%)	62/256 (24.2%)	26/153 (17.0%)	10/95 (10.5%)	164/866 (18.9%)	32.0%	<0.001 ^g^
Married	222/362 (61.3%)	133/256 (52.0%)	91/153 (59.5%)	65/95 (68.4%)	511/866 (59.0%)	47.5%	
Separated	12/362 (3.3%)	8/256 (3.1%)	7/153 (4.6%)	5/95 (5.3%)	32/866 (3.7%)	5.5%	
Divorced	36/362 (9.9%)	37/256 (14.5%)	15/153 (9.8%)	10/95 (10.5%)	98/866 (11.3%)	10.5%	
Widowed	26/362 (7.2%)	16/256 (6.3%)	14/153 (9.2%)	5/95 (5.3%)	61/866 (7.0%)	5.5%	

*p* value compares the patient characteristics from wave to wave using a two-tailed *t* test.

For *Approach #1–4* cells, green is 20% higher than *All Practice* characteristic and orange is 20% lower.

For *All Practices* cells, green is 20% higher than *All Practices in Virginia* and orange is 20% lower.

a Patient level data available for 65 of 76 participating practices.

b Values from the 2021 American Community Survey (census) of Virginia for residents of all ages, except marital status and education which is for residents age 15 years and older [31].

c Comparison of demographic characteristics of A*ll Patients Included in Study* and characteristics of *Virginia Residents*.

d Values derived from chart review of patient’s electronic medical record.

e Values reported by patients on survey.

f Based on 2022 Medicaid and CHIP data [32].

g Fisher exact test was used as Chi-squared was not sufficient.

## Discussion

Our use of real-world data on primary care practices and the communities they care for resulted in a study sample that was highly representative of Virginia and inclusive of most communities in the Commonwealth. While the final sample of 76 practices was below the targeted sample size of 125 practices, it was substantially more practices than others were able to recruit during the COVID pandemic, an especially difficult time to engage any primary care practices in research. More importantly, we were able to demonstrate that changing the routine practice patterns of these 76 practices will have a broad impact throughout the state. Not all practices that were contacted through the four approaches agreed to participate – in fact most declined – but the process expanded our ACORN network and improved our capacity to recruit for future translational studies.

The observed differences in our patient sample versus the demographics of Virginia are largely reflective of the patients seen in primary care and the difference in our study sample (people ages 18–79 years) and the demographic data on Virginia residents which reports on residents of all ages. Older people who are married and widowed are more likely to seek medical care than younger single people. We did not recruit practices in the Veterans Administration or practices so we would expect to have lower proportions of people with Tricare insurance. While we did oversample community health centers for recruitment and purposely targeted practices that served non-English speaking patients in our Targeted Approach, we still had fewer Spanish or non-English speaking patients in our final sample. These practices may take more effort for recruitment into research studies. Our proportion of patients with Medicaid is in line with the proportion of the population with Medicaid at the start of the study, as the number of Virginians with Medicaid doubled between 2019 and 2022 because of Medicaid Expansion [35]. The state rate of people with Medicaid may also be higher because it includes children, who are more likely to be on Medicaid than those ages 18–79 years.

An important part of our equitable recruitment process was knowing not only about the characteristics of the practices we approached for participation but also knowing about the communities they served. In many cases, this had a greater influence on the characteristics of the patients included in the study than the practice characteristics. Knowing about the practices not included in our study sample was also essential to adapting our recruitment processes, which informed us of the people and communities our study could be expected to miss. This helped our team to prioritize new practices to recruit to ACORN, which not only benefitted this study, but also future ACORN efforts. Historically, it is difficult to identify the primary care workforce, let alone know which clinicians practice in which practices [36,37]. Practice-based research networks, like ACORN, have relationships with and know about member practices to create a primary care practice laboratory [38–42], but often do not know about nonmember practices. Our use of licensure and claims data provided an understanding of these nonmember practices.

**Limitations**. A key limitation of the approach that we describe is that it may not be generalizable. Currently, translational scientists do not have universal access to a primary care clinician and practice database. However, the methods we used to create the database could be replicated in other states, particularly by CTS Centers and practice-based research networks that often have connections and an understanding of the communities and primary care practices in their region. Additionally, not all states maintain an APCD like Virginia [43], although other similar data sources could be leveraged in these regions. Additionally, we did not assure representativeness for all people and communities. Our dataset allowed us to address geographic, racial, ethnic, educational, insurance status, and poverty measures for tracking and promoting equitable participation. Future data efforts will better include in research other socially and economically marginalized and medically underserved people and communities.

## Conclusion

Novel use of real-world data as part of a deliberative approach to equitable recruitment of practices for dissemination and implementation trials can ensure inclusion of people and communities that have not historically participated in or benefitted from research. Creating and supporting the use of these data tools should be part of the translational science infrastructure that CTS centers support.
